# Pregnancy has a minimal impact on the acute transcriptional signature to vaccination

**DOI:** 10.1038/s41541-020-0177-6

**Published:** 2020-03-25

**Authors:** John S. Tregoning, January Weiner, Deniz Cizmeci, Danielle Hake, Jeroen Maertzdorf, Stefan H. E. Kaufmann, Geert Leroux-Roels, Cathy Maes, Annelies Aerssens, Anna Calvert, Christine E. Jones

**Affiliations:** 1 Department of Infectious Disease, Imperial College London, St Mary’s Campus, London, W2 1PG UK; 2 Max Planck Institute for Infection Biology, Berlin, Germany; 3 Vaccine Institute, St George’s, University of London, London, UK; 4 Centre for Vaccinology, Ghent University and Ghent University Hospital, Ghent, Belgium; 5 Faculty of Medicine and Institute for Life Sciences, University of Southampton and University Hospital Southampton NHS Foundation Trust, Southampton, UK; 6 Present Address: Core Unit Bioinformatics, Berlin Institute of Health, Berlin, Germany

**Keywords:** Vaccines, Immunology

## Abstract

Vaccination in pregnancy is an effective tool to protect both the mother and infant; vaccines against influenza, pertussis and tetanus are currently recommended. A number of vaccines with a specific indication for use in pregnancy are in development, with the specific aim of providing passive humoral immunity to the newborn child against pathogens responsible for morbidity and mortality in young infants. However, the current understanding about the immune response to vaccination in pregnancy is incomplete. We analysed the effect of pregnancy on early transcriptional responses to vaccination. This type of systems vaccinology approach identifies genes and pathways that are altered in response to vaccination and can be used to understand both the acute inflammation in response to the vaccine and to predict immunogenicity. Pregnant women and mice were immunised with Boostrix-IPV, a multivalent vaccine, which contains three pertussis antigens. Blood was collected from women before and after vaccination and RNA extracted for analysis by microarray. While there were baseline differences between pregnant and non-pregnant women, vaccination induced characteristic patterns of gene expression, with upregulation in interferon response and innate immunity gene modules, independent of pregnancy. We saw similar patterns of responses in both women and mice, supporting the use of mice for preclinical screening of novel maternal vaccines. Using a systems vaccinology approach in pregnancy demonstrated that pregnancy does not affect the initial response to vaccination and that studies in non-pregnant women can provide information about vaccine immunogenicity and potentially safety.

## Introduction

Vaccination of pregnant women (maternal vaccination) can protect both the mother and her offspring from infection^1^. Pregnancy is associated with dynamic adaptions of the immune system throughout gestation to allow immunological tolerance of the developing foetus^2^. During pregnancy, changes in the number and function of immune cells have been observed, with enhanced innate immune responses, as well as reduced numbers of B cells and dendritic cells in the peripheral blood^2–4^. How these differences could impact the response to vaccination in pregnancy is incompletely understood, though recent studies suggest similar antibody responses to influenza and pertussis vaccination in pregnancy^5–7^. Understanding how pregnancy impacts on responses to vaccines is important as new vaccines progress through the vaccine pipeline with a specific indication for use in pregnancy. These vaccines include respiratory syncytial virus (RSV), group B Streptococcus (GBS) and potentially a monovalent pertussis vaccine^8^.

Deeper understanding about the effect of pregnancy on immunity will help to develop and optimise these vaccines, but performing extensive immunological studies in pregnant women is complicated by concerns of risk to mother and foetus. One approach is to use systems vaccinology, which links the transcriptomic (and other ‘omic’) responses to vaccine immunogenicity, efficacy and safety. Systems vaccinology has already led to the identification of innate immune signatures at the individual gene and gene module levels as predictors of vaccine immunogenicity^9,10^.

Systems vaccinology has a number of potential advantages that could accelerate the testing of vaccines^10^. Considerably more data can be generated from fewer volunteers. Depending on the time point that samples are collected, studies can be shorter since the innate response occurs earlier after vaccination. The sampling is relatively non-invasive as large data sets can be determined from a single time point. Critically, systems vaccinology is capable of generating entirely novel avenues of research because the outputs are independent of pre-conceptions: the data are generated and analysed using a non-hypothesis-driven methodology and any differences can then be used to form new hypotheses, which can be tested using other approaches^11^. Systems vaccinology has yet to be applied to immunisation in pregnancy but has the potential to make a significant contribution to this important area of vaccine research especially because of the ability to generate large data sets from smaller numbers of volunteers. The incorporation of animal models into systems vaccinology enables us to address questions that would not otherwise be answerable in clinical studies, particularly with regards to investigating injection sites or developing new formulations.

One important question is how pregnancy alters the early gene transcriptional responses to vaccination. The current study was nested within the Biovacsafe consortium, which had the broader aim of identifying biomarkers of vaccine safety^12^. These transcriptomic profiles, particularly in genes relating to immune function, have been proposed as biomarkers of inflammation after immunisation. In the current study, we investigated the effect of pregnancy on the early response vaccination with Boostrix-IPV, which is used in the UK to boost responses to *Bordetella pertussis* (whooping cough) antigens. It is a multivalent vaccine that also contains diphtheria, tetanus and inactivated polio virus (IPV) antigens. We used RNA microarrays to measure the transcriptomic response in both pregnant mice and women.

## Results

### Vaccination induces a similar response in pregnant and non-pregnant mice

Mice are a widely used preclinical model for understanding the immune response to vaccination. They can be used to examine the early response to vaccination, particularly at the site of immunisation. To model changes after immunisation, we investigated responses in the mouse muscle, the site of immunisation. Ten mice (five pregnant and five non-pregnant controls) were immunised intramuscularly with Boostrix-IPV (produced by GlaxoSmithKline containing diphtheria toxoid, tetanus toxoid, IPV and three *Bordetella pertussis* antigens) and ten mice (five pregnant and five non-pregnant controls) received saline as an injection control. Pregnant mice were time mated and were between 9 and 13 days of pregnancy, which is approximately the second trimester of murine pregnancy (normally 19–21 days long). Muscle samples were collected 24 h after immunisation, and the extracted RNA was analysed by murine microarray analysis.

**Fig. 1:**
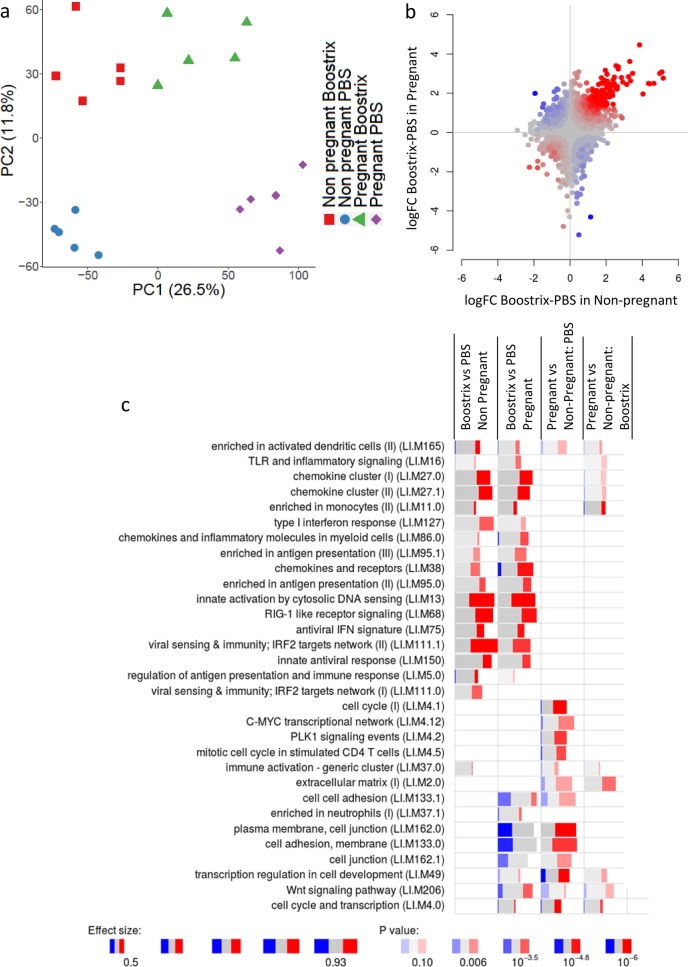
Gene expression in the muscle is comparable 24 h after immunisation between pregnant and non-pregnant mice. Pregnant mice and non-pregnant mice were intramuscularly immunised with Boostrix or PBS. Muscle tissue was extracted at 24 h after immunisation and RNA extracted for analysis by microarray. **a** Principal component analysis (PCA) of gene expression. **b** Concordance/discordance (disco) plots between comparisons in mouse data. Red colour indicates strong concordance (genes regulated in the same direction); blue colour indicates strong discordance (genes regulated in opposite directions). **c** Gene set enrichment analysis of signature in the mouse muscle 24 h after immunisation. Bar sizes correspond to effect size in the enrichment and the intensity of the colour to the *p* value of enrichment. Red and blue boxes indicate the fractions of genes that have, respectively, a significantly higher or lower expression in the test group compared to the non-pregnant group. *N* = 5 per group.

Initial analysis was performed using principal component analysis (PCA), where the dimensionality of a large data set is reduced to just two variables (the principal components) to facilitate the interpretation of complex data sets while minimising data loss. PCA suggested differences between the overall transcriptomic response between the pregnant and non-pregnant mice after immunisation with Boostrix (Fig. 1a) and significant differences between pregnant and non-pregnant animals injected with phosphate-buffered saline (PBS). The experimental design for murine array analysis compared two factors: pregnancy status (pregnant/non-pregnant control) and vaccination status (Boostrix-IPV/placebo [PBS]). To further investigate whether pregnancy altered the global transcriptomic response to vaccination, we conducted discordance/concordance analysis using the disco R package^13^. The idea is that a heuristic score combines the effect size estimates (log_2_ fold changes) and the *p* values between two comparisons, thus providing a measure that corresponds to concordance (when two genes are regulated in the same direction) or discordance (when the two genes are regulated in opposing directions). This shows that the response to a vaccine is largely similar between pregnant mice and non-pregnant control mice, with strong levels of concordance and low levels of discordance (Fig. 1b). The difference to PCA is that disco analysis is at an individual gene level, so it provides a more detailed level of analysis.

To drill down into the response, we investigated modules that had significant enrichment after vaccination (Fig. 1c). Vaccination induced significant enrichment in several modules corresponding to the interferon response (LI.M75, LI.M127 and LI.M150) and innate sensing (LI.M111.1, LI.M13 and LI.M68). These changes were observed in both non-pregnant control and pregnant animals—reflecting the global concordance in changes in the disco analysis. Interestingly, there were significant differences between pregnant and non-pregnant animals 24 h after PBS injection, and these were in clusters relating to cell cycling and adhesion, reflecting the PCA (Fig. 1c, column 3).

The main question was whether the responses to immunisation were different in pregnant and non-pregnant mice. To this end, we have used two approaches. First, we used the disco metric^13^, which allows to compare two comparisons (Fig. 1b). Here we compared the immunisation-related changes in pregnant mice with the changes recorded in non-pregnant mice. While this approach allows visualisation and subsequent gene set enrichment analysis, it does not provide per-gene *p* values. Second, we directly interrogated the interaction term of the linear model for each gene separately, thus obtaining both per-gene *p* values and a gene set enrichment (Fig. 1c). Neither of these approaches showed a significant effect of pregnancy on how the mice reacted to immunisation.

**Fig. 2:**
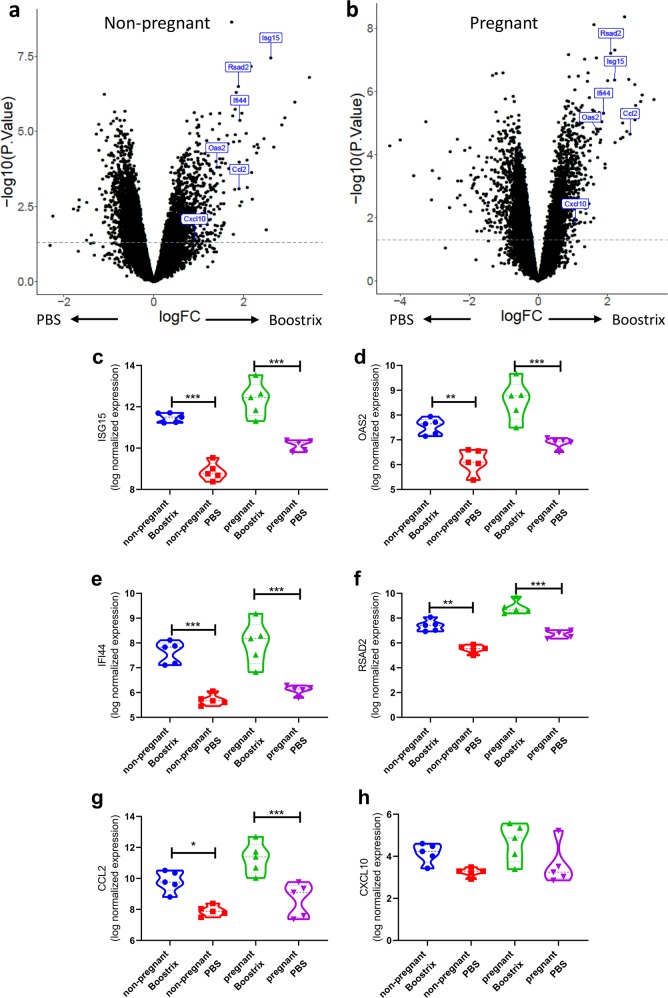
Individual mouse genes in response to vaccination in pregnancy. Differential gene expression analysis comparing non-pregnant mice (**a**) or pregnant (**b**) animals for all genes. Expression of individual differentially expressed genes (**c**–**h**), points represent individual animals, thick dotted line represents median and thin dotted line represents quartiles. *N* = 5 per group. ***p < 0.001, **p < 0.01, *p < 0.05 by ANOVA and post-test.

Overall, there were 902 genes (351 upregulated, 551 downregulated: *q*-value < 0.05) differentially expressed between vaccinated and PBS-treated non-pregnant animals (Fig. 2a) and 1559 genes (685 upregulated, 874 downregulated: *q*-value < 0.05) differentially expressed in pregnant animals following Boostrix-IPV vaccination (Fig. 2b). Previous systems vaccinology studies investigating the response to vaccination with yellow fever vaccine^11^ or inactivated influenza vaccine^14^ have observed a significant upregulation of a number of interferon-stimulated genes (ISG). We selected six individual genes that were differentially expressed after immunisation in a range of other studies (ISG15, OAS2, IFI44, RSAD2, C-X-C chemokine motif ligand 10 (CXCL10) and C-C chemokine motif ligand 2 (CCL2)). We observed significant increases in ISG15 (Fig. 2c), OAS2 (Fig. 2d), IFI44 (Fig. 2e) RSAD2 (Fig. 2f) and CCL2 (Fig. 2g) after immunisation in both pregnant and non-pregnant mice; however, there was no significant difference between Boostrix-IPV vaccinated pregnant and non-pregnant mice. No significant difference was seen in CXCL10 (Fig. 2h) after vaccination in pregnant or non-pregnant mice. This data suggests that vaccination induces a similar early response in both pregnant mice as well as non-pregnant mice.

### Vaccination induces a similar response in pregnant and non-pregnant women

Having observed that immunisation with Boostrix-IPV induces a similar response in pregnant mice to non-pregnant mice, we investigated the response in pregnant women. Thirty women at 16–32 gestational weeks were recruited at St George’s Hospital (London, UK) and immunised with Boostrix-IPV (Table 1). Blood samples were collected into PAXgene tubes immediately prior to vaccination and 24 h later (range from 19 h 19 min to 26 h 40 min). This study was a nested study within a larger study exploring signatures of vaccine safety (BioVacSafe^12^). As a control, RNA transcriptomic data from age-matched non-pregnant women was used. Non-pregnant volunteers had been immunised with Boostrix (Ghent, Belgium), had blood drawn at the same time points and their samples were analysed on the same microarray platform.

**Table 1:** Characteristics of the study populations.

Characteristic	Pregnant (*n* = 30)	Control (*n* = 100)	*p* value
Age, mean (SD)	33.2 (4.7)	27.0 (6.2)	***
Ethnicity, *n* (% Caucasian)	24 (80%)	99 (99%)	***
Approximate gestational age at the time of immunisation (median ± range)	23 weeks (19–32)	N/A	

*N/A* not available.

***p < 0.001.

**Fig. 3:**
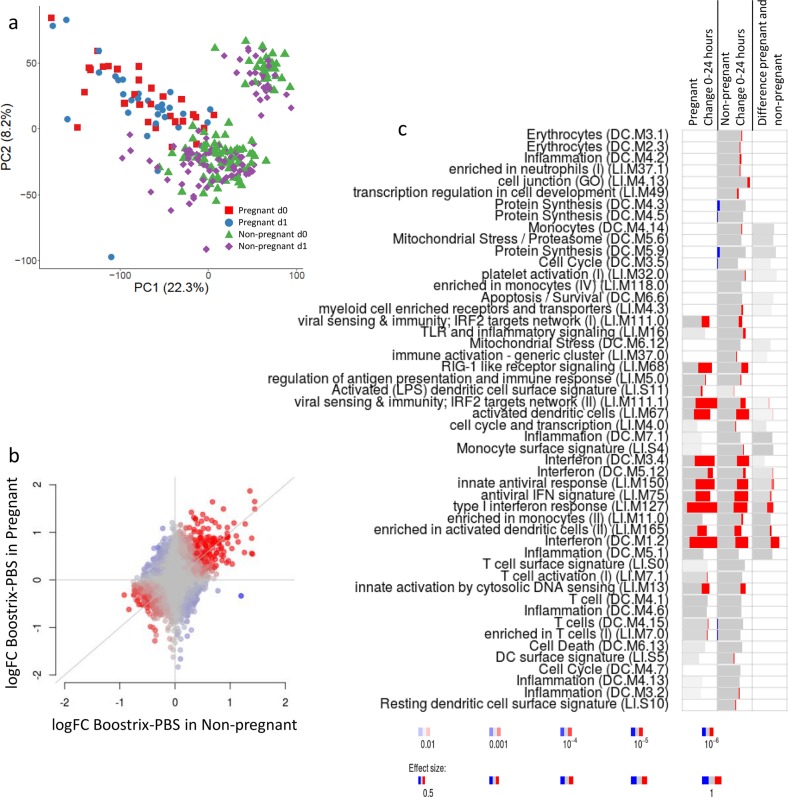
Gene expression in blood is comparable 24 h after immunisation between pregnant and non-pregnant women. Pregnant or non-pregnant women were immunised with Boostrix, blood samples were collected at baseline (d0) and 24 h after immunisation (d1) and RNA was extracted for analysis by microarray. **a** Principal component analysis (PCA) of whole-gene analysis. **b** Concordance/discordance (disco) plots between comparisons in human data. Red colour indicates strong concordance (genes regulated in the same direction); blue colour indicates strong discordance (genes regulated in opposite directions). **c** Gene set enrichment analysis of signature in blood 24 h after immunisation. Bar sizes correspond to effect size in the enrichment, and the intensity of the colour to the *p* value of enrichment. Red and blue boxes indicate the fractions of genes that have, respectively, a significantly higher or lower expression in the group of pregnant women (*n* = 30) compared to the non-pregnant group of women (*n* = 100).

We repeated the analytical approach used in mice, initially taking a global overview of the gene expression changes after immunisation. PCA revealed an overlap between all samples, both before and after vaccination (Fig. 3a). As seen with the mouse data, using PCA, there was some separation between pregnant and non-pregnant women: curiously, the non-pregnant group were heterogeneous, both before and after vaccination, for reasons that are unclear. To investigate whether pregnancy altered the global transcriptomic response to vaccination, we conducted discordance/concordance analysis using the DISCO module^13^. This analysis indicated that the reactions to vaccination are largely similar between non-pregnant and pregnant individuals (Fig. 3b). To investigate whether there were overall patterns in gene expression after immunisation, we used bulk gene set enrichment analysis. Comparing gene expression before and after vaccination, significant increases were seen in similar modules to the mouse, including modules corresponding to interferon response (LI.M75, LI.M127 and LI.M150) and innate sensing (LI.M111.1, LI.M13 and LI.M68), in both pregnant and non-pregnant vaccinated women (Fig. 3c). The interferon module (DC.M1.2) was upregulated in both groups and slightly larger in the pregnant group after vaccination than in the non-pregnant women, which suggests that the interferon response was stronger in the pregnant women.

**Fig. 4:**
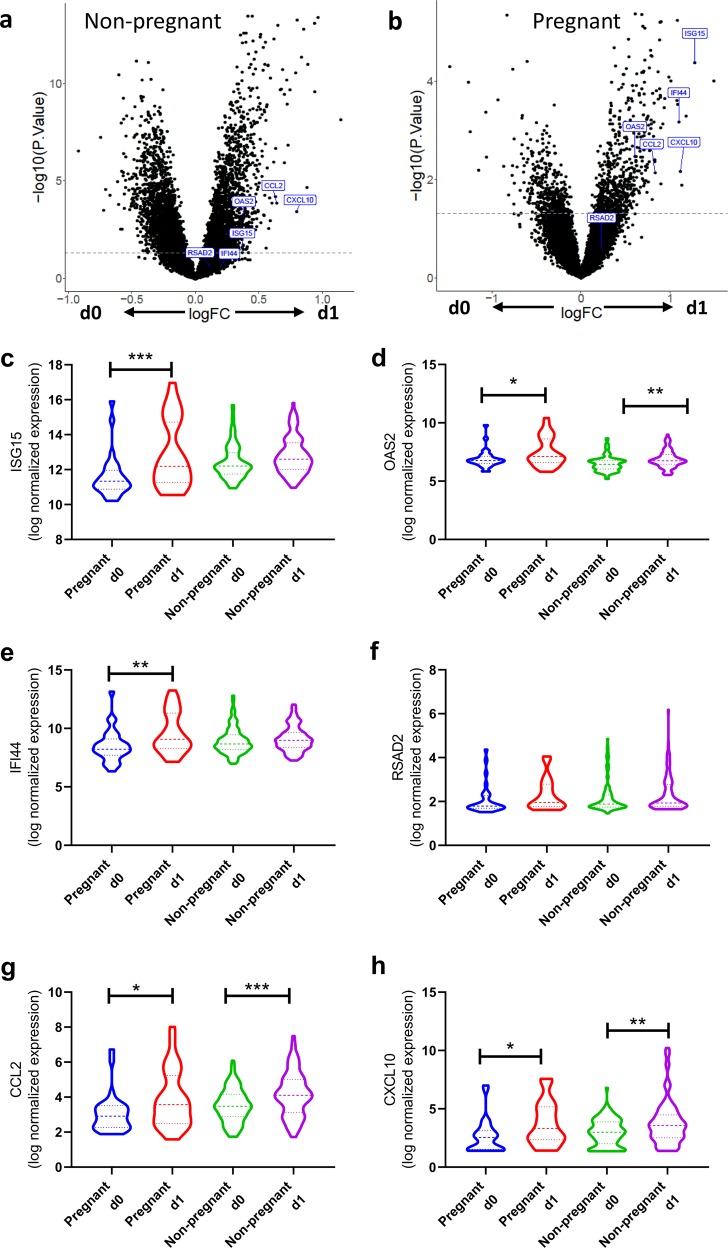
Immunisation induces ISG, regardless of pregnancy status. Differential gene expression analysis comparing non-pregnant (**a**) or pregnant (**b**) women. Individual differentially expressed genes (**c**–**h**), thick dotted line represents median and thin dotted line represents quartile. *N* = 30 in the pregnant group, *N* = 100 in the non-pregnant group. ***p < 0.001, **p < 0.01, *p < 0.05 by ANOVA and post-test.

The gene set enrichment values were reflected in the individual genes that were members of these gene sets with significant fold changes. Overall, there were 2944 (1464 upregulated; 1480 downregulated; Benjamini–Hochberg (BH)-adjusted *p* value < 0.05) genes in response to immunisation with significant differential expression before and after vaccination in non-pregnant women (Fig. 4a) and 46 genes (41 upregulated; 5 downregulated; BH-adjusted *p* value < 0.05) following immunisation in pregnant women (Fig. 4b). We focussed on the same individual ISGs investigated in the mouse study. We observed significant increases in ISG15 (Fig. 4c), OAS2 (Fig. 4d), IFI44 (Fig. 4e), CCL2 (Fig. 4g) and CXCL10 (Fig. 4h) after immunisation in pregnancy, but no change in RSAD2 (Fig. 4f).

While we did not analyse the immune responses to the vaccines, a recent study has looked at the transcript levels as baseline predictors of immunogenicity^15^. When comparing the expression of the suggested marker genes from the published study in pregnancy, we observed the following: among the genes that were associated with a higher response when upregulated, the genes ARRB1, DPP3 and ACTB were significantly higher expressed in non-pregnant compared to pregnant women; there was no difference in expression for MVP, PLEKHB2 or ARPC4 genes and gene expression for GRB2 and RAB24 was higher in pregnant women. Interestingly, four of the six genes that had significantly reduced expression in high responders had lower expression in pregnancy (Supplementary Fig. 1). Overall, vaccination induced a very similar transcriptomic response at 24 h following Boostrix-IPV vaccination in pregnant women and non-pregnant women, as seen in the murine study.

### Of mice and women

**Fig. 5:**
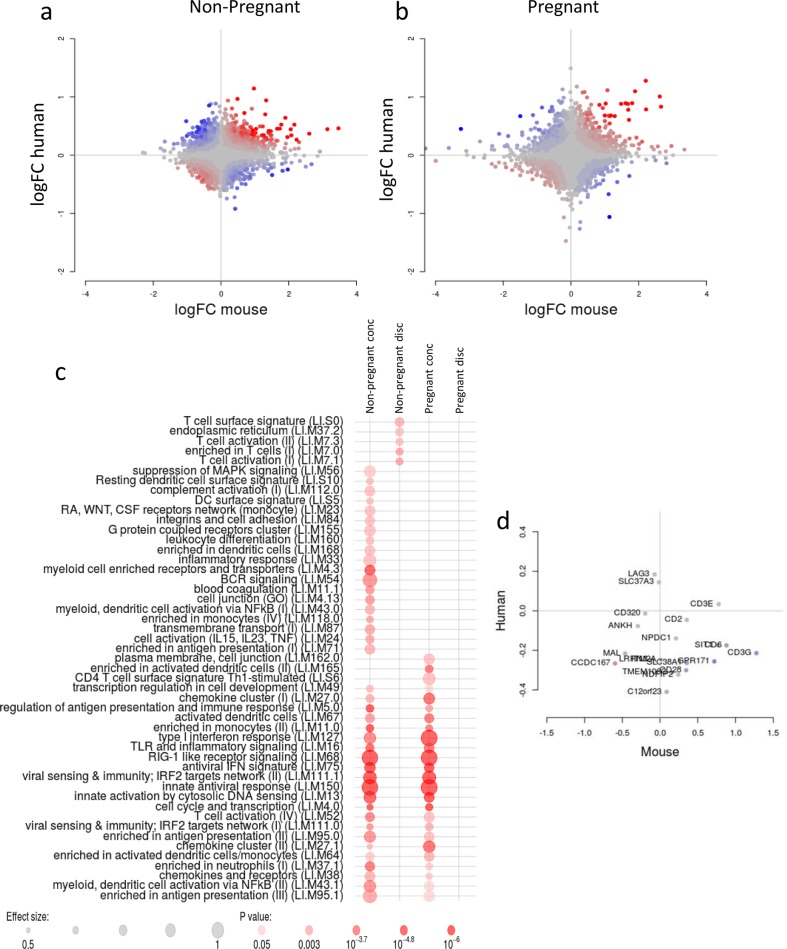
Responses to vaccination during pregnancy are similar in humans and mice. Discordance/concordance plots human vs mouse. Each dot corresponds to a single pair of orthologues. Horizontal axes show the log2 fold change in the tested comparison in murine data. Vertical axes show the log2 fold change in the tested comparison in human data. Colours correspond to the discordance/concordance (disco) score. Red colour indicates high concordance between species and blue colour indicates high discordance between species, i.e. genes regulated in opposite directions. **a** Non-pregnant individuals; **b** pregnant individuals; **c** comparison of interactions between pregnancy and vaccination in murine and human data. **d** Fold change between unvaccinated and vaccinated samples in mouse (*X*-axis) and human (*Y*-axis) samples.

Since the human and mouse samples were analysed using a similar microarray platform, it was possible to compare responses to determine how predictive preclinical mouse models might be for human responses to vaccination. While a direct statistical comparison would not be appropriate, we used the discordance/concordance method, which was developed specifically for comparison between data sets from different species^13^, combined with gene set enrichment analysis. The responses observed in mice were largely concordant with the responses in humans. This similarity in responses was seen in both the non-pregnant (Fig. 5a) and pregnant groups (Fig. 5b). The concordant genes were enriched in the interferon response (LI.M75, LI.M127 and LI.M150) and innate sensing (LI.M111.1, LI.M13 and LI.M68) modules (Fig. 5c, columns 1 and 3). One interesting finding was that a number of T cell activity-related modules were discordant between non-pregnant mice and non-pregnant women (Fig. 5c, column 2). This was driven by several T cell-related genes: indeed, a few of these genes show discordant behaviour between mouse and human samples (Fig. 5d): while the vaccination appeared to lower the expression of genes such as CD3G and GPR171 in humans, in mice the effect was opposite. This finding is not unlike previous comparisons of transcriptomic responses between mice and humans^16,17^.

## Discussion

The current study investigated the effect of pregnancy on the transcriptomic response after vaccination using mouse models and blood collected during clinical studies in humans. Vaccination induced significant upregulation of a number of genes, many of which were in modules associated with innate immunity. We saw a minor impact of pregnancy on the response to vaccination at the global, module and individual gene level. The predominant signal 24 h after vaccination was of innate immune responses, with multiple modules associated with viral sensing and type I interferons. It is also of note that there were broad similarities between the response in pregnant women and mice after vaccination. These data suggest that the immediate immune response to a pertussis-containing vaccine is not affected by pregnancy.

In this study, we investigated the effect of pregnancy on gene expression after vaccination. It was somewhat surprising that the responses to immunisation were similar in pregnancy and in non-pregnant state in both mice and humans. Historically, it was viewed that pregnancy is a period of immune modulation in order to avoid rejection of the foetus, which expresses antigens foreign to the maternal immune system. But this view has been challenged by a number of studies, and it is now clear that this is a simplistic view, and there are complex immune interactions between the foetus and the mother^18^. What we see here is a robust upregulation of innate immune response genes, particularly in the ISG family, both in pregnancy and in non-pregnant mice and humans. It has been proposed that innate immunity may be augmented to compensate for modulated cellular acquired immunity^19^. Alternatively, it is possible that there is local suppression in the female genital tract and decidua at the feto-maternal border during pregnancy, which we did not investigate as we focussed on the systemic responses in blood after intramuscular immunisation.

The individual genes with observed fold changes are likely to be markers of vaccine-induced inflammation. While the study design did not allow us to investigate the link between the gene response and efficacy or immunogenicity, in previous systems vaccinology studies where a link between gene signatures and vaccine-specific immune responses have been explored, changes were observed in similar genes^11,14,20–22^. The similar patterns of gene expression after vaccination to previous studies and the lack of difference between pregnant and non-pregnant individuals supports the observations that pregnancy does not alter the immune response to vaccination^5–7^. We also determined the baseline transcriptome prior to vaccination: previous studies have investigated whether there is a link between baseline gene signatures and immunogenicity. When the gene signature in the current study was compared to a meta-analysis of influenza vaccine studies^15^, we did not see a consistent difference between pregnant and non-pregnant women in genes that were associated with increased expression in high responders to the vaccine. Interestingly, of the genes with reduced expression associated with high responders measured in the current study, PTPN22, PURA, CASP6 and PPIB all had lower levels of expression in pregnancy. The mechanistic impact of these genes on immunogenicity remains to be established. In addition to looking at efficacy, induced gene sets can be used as a measure of inflammation^23^. The similar magnitude of response between the non-pregnant and pregnant groups also indicates that there is a similar level of inflammation in response to vaccination, which suggests that there is no specific signal from this data to suggest an impact on safety associated with vaccination in pregnancy.

Systems vaccinology has largely been harnessed for design of signatures of vaccine immunogenicity. Here we were also interested in the inflammatory transcriptomic profile in the context of safety. Numerous studies have demonstrated the safety of vaccination during pregnancy^24,25^. A recently published study^26^ from the same consortium (Biovacsafe) using the same analytical approaches observed that the chemokines CXCL10 and CCL2 were associated with vaccine-induced inflammation. We found elevated levels of both of these gene transcripts after vaccination, but there was no significant difference between pregnant and non-pregnant individuals in either mouse or humans, suggesting that there was no elevated inflammation that could lead to increased reactogenicity in pregnancy. Here we saw strong concurrence between mouse and human responses to vaccination during pregnancy. Note that we were comparing mouse muscle, the site of injection, with human blood, which is a surrogate measure. Yet, similar profiles were seen in the blood and the muscle, suggesting that the blood profile informs us about what is happening locally at the injection site. Similar patterns favour the mouse model as a predictive tool for understanding inflammatory responses to vaccines in different conditions. While the acute response to vaccination in the mouse closely reflected that seen after human vaccination, there were some differences seen, particularly in modules related to T cells. This reflects the difference in immune experience between humans and experimental (specific pathogen free) mice. Whether through vaccination or infection, adult humans are not naive for most of the vaccine components in Boostrix-IPV, which may explain why there were more T cell signatures in the blood. The gene modules that we observed to be differentially upregulated in response to Boostrix were recently shown to be upregulated in response to adjuvanted influenza vaccine^23^, suggesting that there might be a general response to injected/inactivated vaccines; though it was of note that in the same study responses yellow fever vaccine, a live attenuated virus, had a different kinetic.

This study was nested within a larger consortium (BioVacSafe), which was established to investigate transcriptomic profiles of vaccine safety. The Vaccination In Pregnancy (VIP) study was added on after the main body of the study was performed. It is of note that the two clinical studies were performed at different times, in different locations. There was also a slight difference in the vaccine used: the pregnant, UK cohort received Boostrix-IPV and the non-pregnant Belgian cohort received Boostrix (no-IPV). The difference in vaccine composure does not appear to have made a difference in overall responses at 24 h. The mice used in the study were in their middle trimester of pregnancy, similar as the women; however, there will be differences as murine pregnancies are so much quicker, which may affect interpretation of the data.

It was striking that the innate response to vaccination was not affected by pregnancy. This is important because strategies to boost immune responses to vaccines, for example through adjuvants, may therefore be equally effective and safe in the context of vaccination during pregnancy. The data suggests that, at least in the context of early responses and vaccine safety, studies in healthy non-pregnant women provide useful and correct information about what can occur in pregnant women. Given the pipeline of new vaccines targeted for use in pregnancy, including RSV, GBS and monovalent pertussis^8^, tools that provide greater understanding about the immune response to vaccines in pregnancy are critical. Since acute gene responses are similar, immunological insight derived from systems vaccinology studies in non-pregnant individuals is applicable to pregnant individuals thus accelerating the vaccine pipeline.

## Methods

### The vaccine: Boostrix-IPV

Boostrix-IPV vaccine was used in mice and pregnant women. This vaccine contains pertussis toxin (8 μg), filamentous haemagglutinin (8 μg) and pertactin (2.5 μg) as well as diphtheria toxoid (not less than 2 international units), tetanus toxoids (not less than 20 international units) and IPV types 1–3 (type 1 40 D-antigen unit, type 2 8 D-antigen unit, type 3 32 D-antigen unit). Non-pregnant women received Boostrix, which contains the same components as Boostrix-IPV at the same quantities but without IPV.

### Ethics statement

The animal studies were approved by the Ethical Review Board of Imperial College London, where the experiments were carried out and work was performed in strict compliance with project and personal animal experimentation licences granted by the UK government in accordance with the Animals in Scientific Procedures Act (1986). There was a detailed protocol in place, as required by the humane endpoints described in the animal licence, for early euthanasia in the event of onset of illness or significant deterioration in condition. At the end of the experiment, all animals were culled by cervical dislocation and death confirmed before necropsy. Food and water were supplied ad libitum.

The human study involving pregnant women (VIP gene signature: VIP signature study) was approved by the London-Dulwich Research Ethics Committee (17/LO/0698) and the NHS Health Research Authority. ClinicalTrials.gov: NCT03284515.

Non-pregnant women were recruited in the context of another Biovacsafe study (ClinicalTrials.gov: NCT02555540) that was approved by the Ethics Committee of the Ghent University Hospital (2015/0693).

In both human studies, written informed consent was obtained from all human participants.

### Animals, immunisation and sampling

BALB/c mice of 6–8 weeks of age were purchased from Charles River (Southampton, UK). Three female mice were housed with a single male, which was then boxed out after 3 days. Females were checked for vaginal plugs as an indicator of pregnancy and immunised 13 days after the male was introduced: animals were therefore between 9 (E9) and 13 (E13) days of pregnancy. Ten age-matched non-pregnant female mice were used as controls. Pregnancy was confirmed post-mortem. Animals received a single 50 μl injection of Boostrix-IPV (equivalent to 1/10th of a human dose^27^) or PBS in their right hind leg quadricep muscle and were culled 24 h after the immunisation. There were five female mice in each group (pregnant/non-pregnant receiving either Boostrix-IPV or PBS). When each animal was culled, the injected muscle site was harvested and flash frozen in liquid nitrogen.

### Total RNA preparation from tissue samples

Small pieces of mouse muscle tissue (3 mm × 3 mm × 3 mm) were harvested and flash frozen in liquid nitrogen^26^. Total RNA isolation (including microRNA (miRNA) species) was performed using the miRNeasy Mini Kit (Qiagen, UK), as described in the standard protocol for purification of miRNA and total RNA from tissues and cells. RNA was stored at −80 °C until required for microarray hybridisation.

### Whole-genome microarray analysis

Gene expression data were generated from high-quality RNA samples on an Agilent microarray platform (Agilent Technologies). RNA was labelled with a Low Input Quick Amp Labelling Kit (Agilent Technologies) according to the manufacturer’s instructions. Quantity and labelling efficiency were verified before hybridisation to whole-genome 8 × 60 k mouse expression arrays (Agilent design ID 028005) and scanned at 5 μm using an Agilent scanner. Image analysis and data extraction were performed with the Agilent’s Feature Extraction software (version 11.5) to generate the raw expression data.

### Humans

#### Pregnant women: VIP gene signature study

Pregnant women receiving antenatal care at St George’s University Hospitals NHS Foundation Trust who were between 16 and 32 weeks of pregnancy were eligible to participate. Exclusion criteria included having received a pertussis containing vaccine within the last 12 months and contraindications to vaccination according to the ‘Green Book’ Immunisation against Infectious Disease.

After informed consent had been obtained, the first study visit was arranged to take place between 16 and 32 weeks of pregnancy. At this visit, all participants had a blood sample collected into a PAXgene tube followed by administration of 1 dose (0.5 ml) Boostrix-IPV into the deltoid muscle of their non-dominant arm. A second blood sample was collected into a PAXgene tube 24 h after vaccine administration and information was collected about any adverse events that they had experienced.

#### Non-pregnant women

Non-pregnant women were recruited as part of a larger cohort of healthy young adults who were given a single dose of Boostrix (see above) in the deltoid muscle of the non-dominant arm. This study aimed at finding predictive and early markers of vaccine safety. For this survey, RNA isolated from the pre-vaccination and 24 h post-vaccination blood samples, both collected in PAXgene® blood RNA tubes (BD Biosciences), was used.

### Transcriptome analysis/statistics

Data analysis was performed in R version 3.6.0 (2019-04-26). Microarray data were pre-processed, normalised and analysed for differential expression using R package limma v3.41.15^28^. The raw data were first background corrected using the normexp method. Background corrected signals were quantile normalised between arrays. Linear models were fitted using the limma lmFit function. Differential expression was evaluated using the moderated *t*-statistics, and all *p* values were corrected using the BH approach to obtain *q*-values^29^. PCA was carried out using R prcomp function. Genes that are orthologues in mice and humans were assigned using NCBI HomoloGene^30^. Gene set enrichment analysis was performed with R package tmod (version 0.34) using CERNO statistical test^31,32^. We calculated *p* values corrected for multiple testing using the BH procedure and the effect size area under curve of the gene set enrichment for blood transcriptional modules (BTMs) defined by ref. ^21^. Highly concordantly as well as highly discordantly regulated genes between tissues were identified using the method described by Domaszewska et al.^13^. Magnitude of gene expression change (effect size), significance (adj. *p* value) and direction of gene expression change were used to determine the discordance/concordance score (using the R package disco). All scripts and procedures are available online at https://cran.r-project.org/web/packages/disco/index.html. Data on individual genes was plotted using GraphPad Prism 8.0 (GraphPad Software) and analysed for significance by Student’s *t* test or analysis of variance.

### Reporting summary

Further information on research design is available in the Nature Research Reporting Summary linked to this article.

## Supplementary information

Supplementary Figure 1

Reporting Summary

## Data Availability

The transcriptome data are available at GEO (https://www.ncbi.nlm.nih.gov/geo/) as a SuperSeries (GSE144542) with two SubSeries (GSE144218—mouse data and GSE144540—human data). The raw data for other figures are extracted from the transcriptome data and available on request.
