# Supplementary material for: Pregnancy has a minimal impact on the acute transcriptional signature to vaccination
Source: NPJ Vaccines. 2020 Mar 25;5:29. doi: 10.1038/s41541-020-0177-6 (PMC7096498; doi:10.1038/s41541-020-0177-6)
Supplement: Supplementary file 1 — Supplementary Figure 1 [file 41541_2020_177_MOESM1_ESM.pdf]

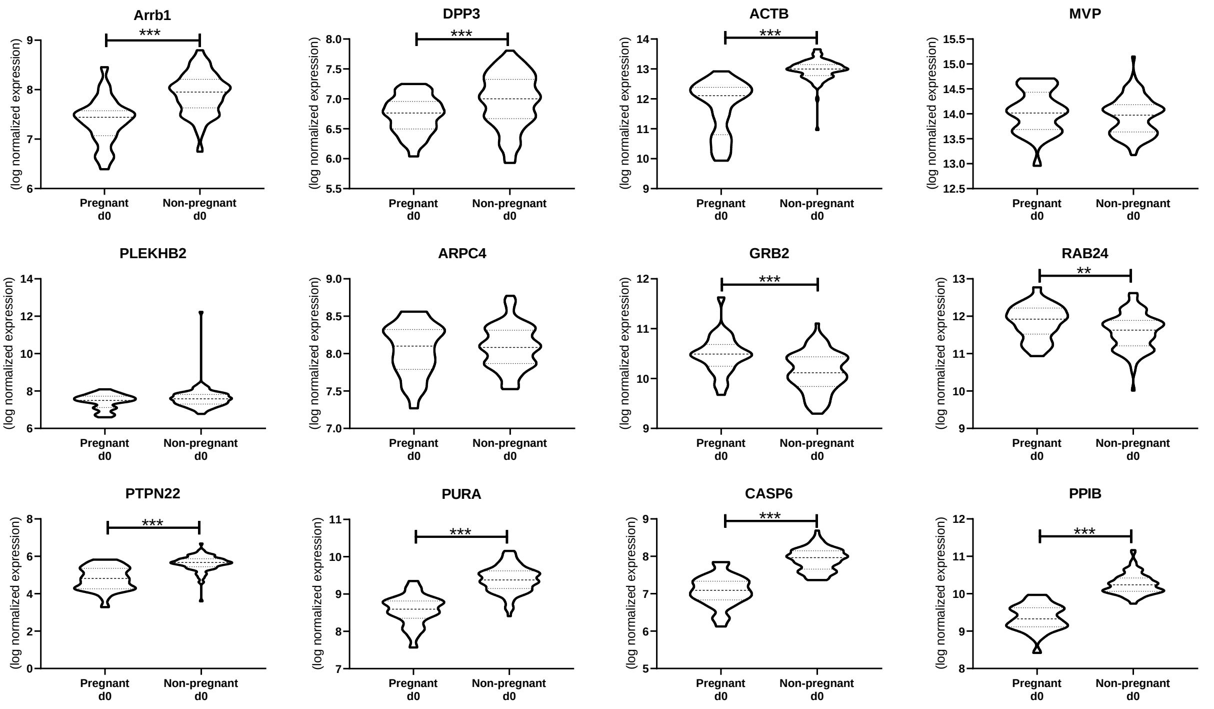

Supplementary figure 1. Effect of pregnancy on expression of potential marker genes. Individual differentially expressed genes, thick dashed line represents median, thin lines represent quartiles.
